# First-time Blood Donors Are Double-edged Swords for Blood Transfusion Centers: A Retrospective Study in Southwest Iran

**DOI:** 10.4274/tjh.galenos.2019.2019.0166

**Published:** 2020-02-20

**Authors:** Hamid Reza Niazkar, Akbar Dorgalaleh, Fariba Rad

**Affiliations:** 1 Gonabad University of Medical Sciences, Student Research Committee, Gonabad, Iran; 2 School of Allied Medical Science, Department of Hematology and Blood Transfusion, Tehran, Iran; 3 Yasuj University of Medical Sciences, Cellular and Molecular Research Center, Yasuj, Iran; 4 Blood Transfusion Research Center, High Institute for Research and Education in Transfusion Medicine, Tehran, Iran

**Keywords:** Transfusion-transmissible infections, Blood donor, Blood donation

## Abstract

**Objective:**

First-time blood donors are the most common group of blood donors. They usually have different motivations for blood donation, some of which provoke the donors to hide risk factors of transfusion-transmissible infections (TTIs). Therefore, detection of TTIs among first-time donors is crucial and can decrease the rate of TTIs among blood recipients. This study aimed to evaluate the prevalence of TTIs among first-time donors in the transfusion center of Kohgiluyeh and Boyer-Ahmad Province (KBTC), Iran.

**Materials and Methods:**

This retrospective study was conducted with volunteer blood donors in 2004-2014 in the KBTC. Various data, including sex, confidential unit exclusion (CUE), previous donation history, and the laboratory findings of confirmatory tests, were extracted from blood donor software. Data were analyzed by SPSS using the chi-square test.

**Results:**

Among 198,501 blood donors, 52,527 (26.46%) were first-time donors, while 145,974 donors (73.54%) were repeat and regular donors. Most of the donors (94.5%) were male, while a minority (5.5%) were female. The CUE option was chosen by 2,237 (1.13%) donors. The incidence of hepatitis B surface antigen (HBsAg) and hepatitis C virus (HCV) was 247 (0.13%) and 134 (0.07%) among the entire study population, respectively. Three donors (0.002%) had confirmed human immunodeficiency virus (HIV), while none of the blood donors were positive for syphilis. Most of the donors with positive HBsAg (95.8%), HCV (86.6%), and HIV (100%) infection were first-time donors.

**Conclusion:**

Since TTIs are more common among first-time blood donors than regular and repeat donors, special considerations should be taken into account for this common group of blood donors.

## Abstract (tr)

**Amaç:**

Kan donörlerinin büyük çoğunluğunu ilk kez kan veren donörler oluşturmaktadır. Bu kişilerin genellikle kan vermek için farklı motivasyonları vardır ve bu da vericilerin transfüzyon ilişkili enfeksiyonları (TTI) saklaması açısından risk oluşturur. Bu nedenle ilk kez verici olanlarda TTI’ları tespit etmek çok önemlidir ve böylece kan alıcılarında TTI’lar azaltılabilir. Bu çalışmada amaç İran’da Kohgiluyeh ve Boyer-Ahmad Bölgesi (KBTC) transfüzyon merkezindeki ilk kez verici olan kan donörlerinde TTI prevelansının araştırılmasıdır.

**Gereç ve Yöntemler:**

Bu çalışma, KBTC’de 2004-2014 yılları arasında gönüllü kan vericilerinin dahil edildiği retrospektif bir çalışmadır. Cinsiyet, gizli ünite dışlaması (CUE), daha önceki kan bağışları, ve doğrulama testlerinin laboratuvar bulguları gibi veriler, kan vericileri yazılımından elde edilmiştir. Elde edilen veriler ki-kare testi kullanılarak SPSS ile değerlendirilmiştir.

**Bulgular:**

198,501 kan vericisinden 52,527’i (%26,46) ilk kez kan veren donörler, 145,974’i ise düzenli olarak kan verenler veya daha önce kan vermis olanlardı (%73,54). Vericilerin çoğu erkekti (%94,5), kadın verciler azınlıktaydı (5,5%). CUE seçeneği 2,237 (%1,13) verici tarafından kullanılmıştı. Bütün çalışma grubunda hepatit B yüzey antijeni (HBsAg) ve hepatit C virüsü (HCV) sıklığı sırasıyla 247 (%0,13) ve 134 (%0,07) idi. Üç vericide doğrulanmış insan bağışıklık yetmezliği virüsü (HIV) bulundu (%0,002), hiç bir hastada sifilis tespit edilmedi. HBsAg (%95,8), HCV (86,6%), ve HIV (100%) enfeksiyonu olan vericilerin çoğunluğu ilk kez kan vericisi olan gruptaydı.

**Sonuç:**

TTI’lar tekrarlayan kez verici olanlar veya düzenli verici olanlarla karşılaştırıldığında ilk kez verici olanlarda daha sık görülmektedir ve bu vericilerin kullanılması durumunda özel önlemlerin alınması gereklidir.

## Introduction

Blood transfusion has a fundamental role in medical services. Although blood donation can improve the quality of patients’ lives, it is one of the main causes of the transmission of viral infections, including hepatitis B virus (HBV), hepatitis C virus (HCV), and human immunodeficiency virus (HIV). Several studies have shown that the chance of transmission of these infections is about 1% in each blood unit transfusion, while a 6.2% chance to transmit hepatitis was also reported [1,2,3,4].

Since blood can only be provided by human resources, transfusion-transmissible infections (TTIs) via these products are important issues. Therefore, blood safety is the most important concern of blood transfusion worldwide [5]. In the process of ensuring blood safety, donors have a crucial role [6]. As a result, standard and relatively strict blood donation conditions are used by blood transfusion centers to improve blood safety. This includes a voluntary blood donation system, a confidential unit exclusion (CUE) system, the exclusion of high-risk donors, and standard and sensitive blood-borne disease detection systems. High-risk donors are those with risky behaviors, such as multiple sexual partners or intravenous drug injection, which can increase the risk of TTIs [7,8,9].

According to the standards of blood transfusion organization, volunteer donors are classified into two groups: first-time donors and repeated and regular donors [10]. Several studies have shown that first-time blood donors are the most common group of blood donors [10]. These donors usually have different motivations for blood donation. Some of these motivations provoke donors not only to hide risk factors during interviews but also to increase the risks of the blood donation system. At the same time, studies in different communities have shown that the prevalence of TTIs in first-time donors is higher than that of other donors [10]. Therefore, evaluation and detection of TTIs in first-time donors are crucial and can decrease the rate of TTIs among blood recipients.

Despite various strategies and significant advances in the detection and diagnosis of these diseases and the reduction of the window period by new-generation diagnostic kits, TTIs remain the most important challenge in blood transfusion. This study aimed to evaluate the prevalence of TTIs among first-time donors at the transfusion center of Kohgiluyeh and Boyer-Ahmad Province, Iran (KBTC).

## Materials and Methods

This retrospective study was performed with 198,501 volunteer blood donors from 2004 to 2014 at the KBTC. Demographic data including sex and CUE, as well as donation history and laboratory findings of confirmatory tests, were extracted from blood donation software. Blood donors were classified into two groups as first-time donors or repeated and regular donors. A first-time donor is a donor who is donating blood for the first time. A repeated and regular donor is a donor who has donated blood at least once in the past [10].

The results of HIV, HBV, HCV, and syphilis tests were also extracted. The screening tests were performed with commercially available enzyme-linked immunosorbent assay kits.

Hepatitis B surface antigen (HBsAg) was checked with Behring (Marburg, Germany), Siemens (Marburg, Germany), and Bio-Rad (Hercules, CA, USA) kits; anti-HCV was checked with BioMerieux (Marcy l’Etoile, France) and Avicenna (Avicenna Medical Center, Moscow, Russia) kits; and HIV-Ab was tested with BioMerieux (Marcy l’Etoile, France), Bio-Rad (Hercules, CA, USA), and Adaltis (Montreal, Canada) kits. All repeatedly reactive results were confirmed by a neutralization test (Behring, Marburg, Germany) for HBV, a recombinant immunoblot assay for HCV (Inonogenetic, Ghent, Belgium), or a western blot assay for HIV (Inonogenetic, Ghent, Belgium).

According to Iranian Blood Transfusion Organization (IBTO) policies, each blood unit with positive results confirmed for blood-borne diseases was excluded and the corresponding donor was recalled for counseling and appropriate treatment.

The rate of confirmed positive HBV, HCV, and HIV tests was compared between the studied groups. The frequencies of HBsAg, HIV, and HCV and 95% confidence intervals (CIs) were calculated using SPSS software. The prevalence rate of infections was calculated for each group and compared using the chi-square test, and differences were considered significant at p<0.05. This study was approved by the ethic committee of Yasuj University of Medical Sciences (IR.Yuma.rec.1396.22).

## Results

A total of 198,501 blood donations were performed from 2004 to 2014 at the KBTC. These included 52,527 (26.46%) first-time donors and 145,974 (73.5%) repeated and regular donors. Out of the total of 198,501, 187,691 donors (94.5%) were male and 10,810 donors (5.5%) were female (17.1:1). The CUE option was chosen by 2,237 (1.13%) donors, while 196,264 (98.87%) of the volunteer donors did not choose the CUE option and were thus considered as the CUE-negative group. According to the history of donation, the distribution of blood donors in CUE groups is presented in Table 1.

Among the 198,501 blood donors, 247 (0.13%) and 134 (0.07%) donors were positive for HBsAg and HCV, respectively. HBsAg had higher prevalence compared to HCV infection in the entire study population [0.13% and 0.07%, respectively; odds ratio (OR)=1.84; 95% CI=1.49-2.27; p<0.0001]. Three donors (0.002%) had confirmed HIV and none of the blood donors were positive for syphilis. In addition, concomitant infections were not detected in any donor.

Among the 52,527 first-time blood donors, 231 (0.44%), 116 (0.2%), and 3 (0.005%) donors were positive for HBsAg, HCV, and HIV, respectively. HBsAg had a higher prevalence than HCV infection among first-time donors (OR=1.98; 95% CI=1.59-2.48; p<0.0001).

In the population studied in this investigation, 1.08% (24/2,237) of CUE-positive and 0.18% (357/196,264) of CUE-negative donors were positive for disease markers. Table 2 shows a significantly higher prevalence of HBsAg and HCV in the CUE-positive than CUE-negative donors (1.08% and 0.18%, respectively; OR=5.84; 95% CI=3.875-8.820; p<0.0001).

The prevalence of confirmed HBsAg was 1.32% (14/1,060) and 0.42% (217/51,467) among the first-time CUE-positive and first-time CUE-negative donors, respectively (OR=3.1; 95% CI=1.814-5.312; p<0.001). The prevalence of confirmed HCV was 0.85% (9/1,060) and 0.21% (107/51,467) among the first-time CUE-positive and first-time CUE-negative donors, respectively (OR=4.05; 95% CI=2.060-7.990; p<0.0001) (Table 3). There were 3 HIV-positive donors among the CUE-negative first-time donors.

A significantly higher prevalence of HBsAg and HCV infection was observed in male donors than females among the first-time donors (p<0.001) (Table 4).

## Discussion

Blood and its components are among the most important causes of TTI transmission. The possibility of TTI transmission in the transfusion of every blood unit is about 1% [6]. This is a relatively high rate for transmission of blood-borne diseases because some of these infections are severe, life-endangering ones that are incurable or have a difficult treatment process [6,7]. Thus, TTIs are a significant challenge for blood transfusion services worldwide and require precise precautions. Different factors such as vaccination programs, high-risk behaviors, and the socioeconomic status of people can affect the risk of TTIs in any community. In recent decades vaccination against HBV significantly decreased the rate of TTIs in different countries. In Iran, vaccination against HBV significantly decreased the rate of HBV infections in comparison with countries without this program or those with late establishment. The incidence of HBV, HCV, and HIV is also higher in low-income countries than in middle- and high-income countries. These data show that there is a direct correlation between the economic condition of countries and TTI incidence. Those countries with higher income can more easily provide preventive and vaccination programs for their people than countries with lower income [11,12]. Education is another important factor that can significantly decrease the rate of TTIs among blood donors, mostly by reducing risky behaviors [13].

Due to the crucial role of blood safety in blood transfusion services, huge efforts are made to improve the safety of blood and its components [1,2,6,10,14]. These efforts are performed in different stages of blood transfusion processes, from blood donor selection to blood release [3,10,15]. Donor selection is an important step in blood safety and different studies have revealed that suitable and appropriate donor selection can significantly improve blood safety [15,16]. It was shown that blood components of repeated and regular blood donors have a lower risk of TTI transmission than those of first-time donors [10,17,18,19,20]. On the other hand, it has been shown that first-time donors are the most common donors in blood transfusion centers [10,18]. These two issues highlight the importance of the donor selection process in blood transfusion centers, which can significantly improve the safety of blood and blood components. In our study, about one-third of the donors were first-time donors. Similar results were observed in several other studies in Iran and other countries [15,17,18].

In our study, similar to many others, it was revealed that TTIs are more common among first-time donors than repeated and regular donors [6,15]. The CUE system is a commonly used system in most blood transfusion centers. However, its usefulness is questionable based on a considerable number of studies [21,22,23]. Despite this issue, the IBTO has used this system to improve the safety of blood and its components [15,24]. Several studies confirmed that CUE is a relatively cost-beneficial system that can significantly improve the safety of blood products [15,25]. In our study, this issue was observed and the CUE option was more commonly used by first-time donors. In our study, about 2% of the first-time donors used the CUE option (CUE-positive), while only 0.8% of repeated and regular donors did. This rate of CUE positivity among the first-time donors in our study is lower than that in the study of Vogler et al. [26], who reported about 5% CUE positivity among their first-time donors.

Moreover, a significantly higher prevalence of TTIs was observed among first-time donors with positive CUE in comparison with the CUE-negative first-time donors. The higher prevalence of TTIs among the first-time donors and the CUE-positive first-time donors is in agreement with other studies conducted in Iran, Australia, the United Kingdom, and the Netherlands [4,15,17,24,25].

In our study, the prevalence of HBsAg and HCV was 0.13% and 0.07% among blood donors, respectively, and 0.44% and 0.2% in the first-time blood donors, respectively. This rate of infection in the KBTC is lower than those of other studies [1,2,5,27,28,29]. This discrepancy in the prevalence of TTIs in different populations around the world reflects a variety of high-risk behaviors, population risks, health statuses, and selection procedures in those regions.

In our study, the prevalence of HBsAg and HCV infection had increased during 2005-2007 and 2009-2011 among voluntary first-time donors, while Farshadpour et al. [4], Amini Kafi‐Abad et al. [30], and Khodabandehloo et al. [31] reported a decreased trend in the prevalence of HBV and HCV between 2004 and 2012. The reason for this increase in our study could be related to the significant increase in the number of first-time blood donors, which increased from 4,139 in 2005 to 7,031 in 2011. Also, this increase could be related to a combination of other factors including vaccination against HBV; low public knowledge about blood-borne infections and routes of transmission in the past such as traditional tattoos, traditional circumcision, and cupping therapy; and the effectiveness of prospective donor screening measures.

It seems that a higher rate of TTIs among first-time donors is a relatively significant challenge for blood transfusion centers and special policies such as CUE should be considered for these donors to improve the safety of blood and its components.

Similar to other studies, our results showed a higher prevalence of HBsAg compared to HCV in both total and first-time donors [30,32]. This high prevalence may be due to the higher rate of HBV in the general population of this province, whereas this issue was not determined in any other study and consequently further studies are required to confirm this issue [33,34].

## Conclusion

Due to the high rate of TTIs among first-time donors, it is crucial to implement some preventive programs among this common type of blood donors to reduce the overall incidence of TTIs among blood recipients.

## Figures and Tables

**Table 1:**
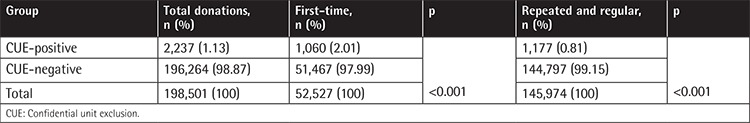
Distribution of blood donors in confidential unit exclusion -positive and confidential unit exclusion-negative groups.

**Table 2:**
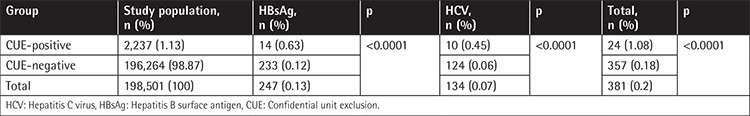
The prevalence of confirmed hepatitis B surface antigen and hepatitis C virus among confidential unit exclusion-positive and confidential unit exclusion-negative groups.

**Table 3:**
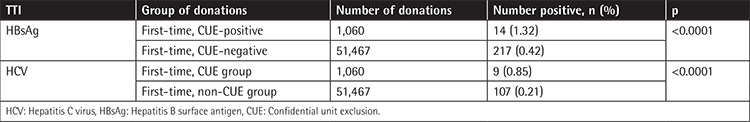
Comparison of hepatitis B surface antigen and hepatitis C virus prevalence among the confidential unit exclusion-positive and confidential unit exclusion-negative first-time donors.

**Table 4:**
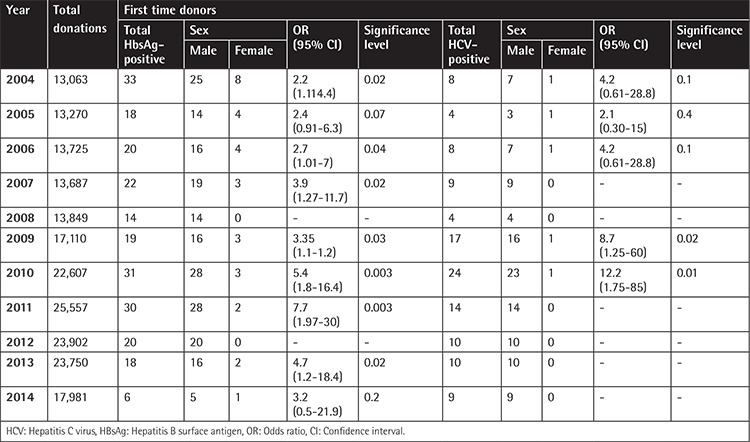
Sex distribution of first-time reactive donors in whole study population.
